# Supplementary material for: ERNIE-UIE: Advancing information extraction in Chinese medical knowledge graph
Source: PLoS One. 2025 May 29;20(5):e0325082. doi: 10.1371/journal.pone.0325082 (PMC12121792; doi:10.1371/journal.pone.0325082)
Supplement: S1 Appendix — (DOCX) [file pone.0325082.s001.docx]

Appendix 1 Code for Parsing Knowledge Extraction Results

# Extract result and parse code

folder_path = './txt'

new_folder_txt = "./output_txt"

new_folder_excel = "./output_excel"

for file in os.listdir(folder_path):

file_path = os.path.join(folder_path, file)

file_name = os.path.splitext(file)[0]

tag = "ie"

new_filename_txt = "{}{}.txt".format(file_name, tag)

new_filename_excel = "{}{}.xlsx".format(file_name, tag)

f = open(file_path, "r",encoding="utf-8")

lines = []

for line in f:

lines.append(line)

f.close()

f = open(os.path.join(new_folder_txt, new_filename_txt),"w",encoding="utf-8")

wb = openpyxl.Workbook()

ws = wb.active

# Write header row

ws["A1"] = "label"

ws["B1"] = "head_entity"

ws["C1"] = "head_prob"

ws["D1"] = "tail_entity"

ws["E1"] = "relation_prob"

ws["F1"] = "relation"

row = 2

for text in lines:

# Call the pre-trained IE program, pass 'text' as a parameter, and get the return value

result = my_ie(text)

# Convert the list to JSON format

result_json = json.dumps(result,indent=4, ensure_ascii=False)

input = json.loads(result_json)

for element in input:

if isinstance(element, dict):

for label, value in element.items():

if isinstance(value, list):

for event_type in value:

head_entity = event_type.get("text")

head_prob = event_type.get("probability")

relations = event_type.get("relations", {})

if relations:

for relation, tail_entities in relations.items():

for tail_entity in tail_entities:

tail_prob = tail_entity.get("probability")

tail_entity = tail_entity.get("text")

ws[f"A{row}"] = label

ws[f"B{row}"] = head_entity

ws[f"C{row}"] = head_prob

ws[f"D{row}"] = tail_entity

ws[f"E{row}"] = tail_prob

ws[f"F{row}"] = relation

row += 1

else:

ws[f"A{row}"] = label

ws[f"B{row}"] = head_entity

ws[f"C{row}"] = head_prob

row += 1

wb.save(os.path.join(new_folder_excel,new_filename_excel))

result = str(result)

f.write(result + "\n")

f.close()

print("Extraction completed. please check the file related to" + new_filename_excel)
