# Supplementary material for: ERNIE-UIE: Advancing information extraction in Chinese medical knowledge graph
Source: PLoS One. 2025 May 29;20(5):e0325082. doi: 10.1371/journal.pone.0325082 (PMC12121792; doi:10.1371/journal.pone.0325082)
Supplement: S2 Appendix — (DOCX) [file pone.0325082.s002.docx]

Appendix 2 Code for Mapping Knowledge Extraction Results

# Extract results and map code

import os

import pandas as pd

# Define mapping relationships from relationships to labels

relation_to_label = {

"is responsible for": "Other Measures","has symptom": "Symptom",

"requires examination": "Examination","requires measure": "Other Measures",

"involves department": "Department","leads to": "Disease",

"involves equipment": "Equipment","involves diet": "Diet",

"involves vital sign": "Vital Sign","treats disease": "Disease",

"treats": "Disease","alleviates": "Symptom",

"involves drug": "Drug","has effect": "Effect"

}

excel_files = [file for file in os.listdir() if file.endswith(".xlsx")]

processed_files = [file for file in excel_files if file.startswith("new_")]

original_files = [file for file in excel_files if file not in processed_files]

# Process each Excel file to be handled

for excel_file in original_files:

df = pd.read_excel(excel_file)

# Get rows with tail entities

tail_entity_rows = df[df['tail_entity'].notnull()]

new_entities = set()

for index, row in tail_entity_rows.iterrows():

tail_entity = row['tail_entity']

head_entities = set(df[(df['tail_entity'].isnull()) & (df['relation_prob'].isnull()) & (df['relation'].isnull())]['head_entity'])

if tail_entity not in head_entities and tail_entity not in new_entities:

relation = row['relation']

label = relation_to_label.get(relation, "unknown label")

new_entities.add(tail_entity)

new_row = {

'label': label,

'head_entity': tail_entity,

'head_prob': None,

'tail_entity': None,

'relation_prob': None,

'relation': None

}

df = df.append(new_row, ignore_index=True)

new_excel_file = "new_" + excel_file

df.to_excel(new_excel_file, index=False)

# Check relationships and verify code

import pandas as pd

df_triple = pd.read_excel('Entities and triplet data.xlsx')

# Correspondence table for Head Entity Label - Relationship - Tail Entity Label

relation_dict = {

('Disease', 'has symptom'): 'Symptom',

('Disease', 'requires examination'): 'Examination',

('Disease', 'requires measure'): 'Other Measures',

('Disease', 'involves department'): 'Department',

('Disease', 'leads to'): 'Disease',

('Disease', 'involves equipment'): 'Equipment',

('Disease', 'involves diet'): 'Diet',

('Examination', 'involves vital sign'): 'Vital Sign',

('Surgical Procedure', 'treats disease'): 'Disease',

('Person', 'is responsible for'): 'Other Measures',

('Drug', 'treats'): 'Disease',

('Drug', 'alleviates'): 'Symptom',

('Other Measures', 'involves drug'): 'Drug',

('Other Measures', 'has effect'): 'Effect'

}

# Verify the triplet data

def check_relation(row):

key = (row['head_entity_label'], row['relation'])

if key in relation_dict and relation_dict[key] == row['tail_entity_label']:

return ''

else:

return 'not match'

df_triple['Verified results'] = df_triple.apply(check_relation, axis=1)

df_triple.to_excel('The verified data.xlsx', index=False)
