# Supplementary material for: ERNIE-UIE: Advancing information extraction in Chinese medical knowledge graph
Source: PLoS One. 2025 May 29;20(5):e0325082. doi: 10.1371/journal.pone.0325082 (PMC12121792; doi:10.1371/journal.pone.0325082)
Supplement: Attachment — Submitted filename: Response to Reviewers.docx [file pone.0325082.s005.docx]

Response to Reviewer’s Comments

Dear Dr. Emiliano Damian Alvarez Leites and Reviewers,

We were pleased to know that our work was rated as potentially acceptable for publication in Journal, subject to adequate revision. We thank the reviewers for the time and effort that they have put into reviewing the previous version of the manuscript. Their suggestions have enabled us to improve our work. Based on the instructions provided in your letter, we uploaded the file of the revised manuscript. Accordingly, we have uploaded a copy of the original manuscript with all the changes highlighted by using the track changes mode in MS Word.

Appended to this letter is our point-by-point response to the comments raised by the reviewers. The comments are reproduced and our responses are given directly afterward in a different color.

We would like also to thank you for allowing us to resubmit a revised copy of the manuscript.

We hope that the revised manuscript is accepted for publication in the Journal of PLOS One.

Sincerely,

Jing Zheng

Journal Requirements:

When submitting your revision, we need you to address these additional requirements.

1. Please ensure that your manuscript meets PLOS ONE's style requirements, including those for file naming. The PLOS ONE style templates can be found at
   https://journals.plos.org/plosone/s/file?id=wjVg/PLOSOne_formatting_sample_main_body.pdf and 
   https://journals.plos.org/plosone/s/file?id=ba62/PLOSOne_formatting_sample_title_authors_affiliations.pdf

**Response:**

We will check carefully to meet the journal's formatting requirements.

2. In your Methods section, please include additional information about your dataset and ensure that you have included a statement specifying whether the collection and analysis method complied with the terms and conditions for the source of the data.

**Response:**

**The data sources statement** **was added in the methods section of the paper(See 3.1 Overall Framework), and a data availability statement was added at the end of the paper.**

**All data used in this study were obtained from publicly available sources. Web scraping was conducted strictly in accordance with the robots.txt protocols of the respective websites, ensuring compliance with their terms of service. Literature-derived data were exclusively sourced from open-access publications and public domain resources. The data collection and analysis methods fully complied with the terms and conditions of the original data sources. Careful attention was paid to respecting intellectual property rights and avoiding any potential copyright infringements. No proprietary or restricted-access data were utilized in this research. The data acquisition and use processes were designed to maintain ethical standards and legal requirements for scientific research.**

**Data Availability Statement**

**The authors confirm that the data supporting the findings of this study are available within the article and its supplementary materials.**

 3. We note that the grant information you provided in the ‘Funding Information’ and ‘Financial Disclosure’ sections do not match.

When you resubmit, please ensure that you provide the correct grant numbers for the awards you received for your study in the ‘Funding Information’ section.

**Response:**

**We have supplemented the information about the funding and indicated it in the cover letter.**

Reviewer #1: This paper explores the application of Universal Information Extraction (UIE) technology, specifically the ERNIE-UIE model, to construct a Chinese medical knowledge graph with minimal annotated data. By integrating ontology modeling, web scraping, and fine-tuning strategies, the study constructs a knowledge graph containing 8,525 entities and 9,522 relationships, which are verified using graph algorithms. The findings demonstrate that this approach effectively addresses the challenge of low-resource knowledge graph construction and enhances efficiency in knowledge extraction and storage.

**Response:**

Thanks for your positive comments. We have carefully revised this manuscript based on the reviewer’s comments. We hope that the revisions and improvements would satisfactorily address the reviewer’s concerns.

1. What the authors claim is a KG, is simply not that. They have a property graph, stored in Neo4J. A Knowledge Graph is a dataset constructed using triples represented in the RDF format (a W3C standard), which are annotated with a RDFS or OWL ontology (both are W3C standards), and are retrievable using the SPARQL query language (another W3C standard). Property graphs are not that.

**Response:**

Thanks for the valuable comments. We carefully searched for relevant information and explained the reasons why neo4j was used.

*Reference:*

*<https://neo4j.com/blog/rdf-vs-property-graphs-knowledge-graphs/>*

RDF-based storage and graph database-based storage are two common knowledge graph storage methods. RDF-based storage uses RDF format to represent triple information, which has the characteristics of flexibility, openness, and semantic expression. The graph database-based storage stores entities and relationships in a graph structure, which has the characteristics of association, fast query, elastic expansion, and complex analysis. In practical applications, the selection of a suitable storage method needs to consider specific application scenarios and requirements. With the development of knowledge graphs, it is believed that these two storage methods will continue to be widely used and bring more convenience and value to the construction and application of knowledge graphs.

Currently neo4j has been widely used in knowledge graphs.

*Added References*

*[14]Yuan D, Zhou K, Yang C. Architecture and Application of Traffic Safety Management Knowledge Graph Based on Neo4j[J]. Sustainability, 2023, 15(12): 9786.*

*[15]Smith K, Liu F, Phanish D, et al. Research Collaboration Discovery through Neo4j Knowledge Graph[M]//Practice and Experience in Advanced Research Computing 2024: Human Powered Computing. 2024: 1-7.*

**(See Reference14,15)**

**Therefore, we added the following relevant content in the paper.**

**Neo4j (a graph database) was the knowledge storage tool selected for this study. It is a high-performance graph database management system that employs graph theory-based storage structures to manage data. Developed by Neo Technology, it is one of the most popular graph databases on the market, particularly well-suited for handling highly interconnected data. Based on the graph data model, Neo4j’s data structure consists of four components: nodes, relationships, properties, and labels. Compared with RDF storage, Neo4j offers significant advantages in terms of graph traversal performance, intuitive data modeling, real-time querying, and tool ecosystem, making it ideal for application scenarios requiring efficient graph operations and real-time responsiveness[14,15].**

**(see 4.3 Knowledge Storage of Hypertensive Intracerebral Hemorrhage)**

2.The authors do not show any ontology being used, they do not show SPARQL queries being used, etc. They treat their constructed "knowledge graph" as a regular graph, using analytics which are common for graphs, not RDF knowledge graphs.

**Response:**

We sincerely appreciate the valuable comments. We have checked the literature carefully, and cited the related content as following.

*Reference:*

*Khan A. Knowledge graphs querying[J]. ACM SIGMOD Record, 2023, 52(2): 18-29.*

A number of technologies exist for KG querying, e.g.,SPARQL, SQL extensions, Datalog, graph query lan guages, keyword query, exampler query, faceted search,visual query, query templates, natural language ques tions, conversational QA, multimodal QA, and interac tive methods (e.g., feedback, explanation, suggestion,autocompletion, etc.).SPARQL is the W3C recommended query language for RDF. Neo4j provides a native graph database with property graph data model and Cypher query language.It also supports the Apache TinkerPop acting as a connectivity layer to use Grem lin. Neo4J supports several graph analytic tools (e.g.,Popoto.js, Neo4j Bloom) that assist in interactive, visual query building and suggestion. Neo4J’s graph data science library implements three graph embedding methods (FastRP, GraphSAGE, and Node2Vec), node classification and regression, link prediction.The top-10 commercial graph databases support various languages for querying of KGs – as RDF triples or property graphs. (Details in the following figure)

**Therefore, our answer to this point is that, Cyper is more appropriate than SPARQL based on the database technology used in this study.**

3.Additionally, the authors continuously use the term "triplets", which is a medical term for describing three siblings being born by the same mother at the same time. What they think about are structures called a "triples", e.g. RDF triples. A triple is a tuple of size three (3-tuple, triple).

**Response:**

We sincerely thank the reviewer for careful reading.

**As suggested by the reviewer, we have corrected the “triplets” into “triple”.**

4.Ultimately, the authors fail to show any meaningful reason why they converted the data into a Neo4j graph structure, when they could've simply loaded the parsed data into a relational database or a Python data structure and do the analysis they set out to do.

**Response:**

Thanks for the valuable comments.Based the comments, we have carefully reviewed some articles,and cited the related content as following.

*Reference:*

*Vicknair C, Macias M, Zhao Z, et al. A comparison of a graph database and a relational database: a data provenance perspective[C]//Proceedings of the 48th annual Southeast regional conference. 2010: 1-6.*

“Both systems performed acceptably on the objective benchmark tests. In general, the graph database did better at the structural type queries than the relational database. In full-text character searches, the graph databses performed significantly better than the relational database.”

Neo4j has advantages over RDF storage or SQL and other relational database storage.The advantage of graph databases is that they can naturally represent knowledge graph structures. The nodes in the graph represent objects in the knowledge graph, and the edges in the graph represent object relationships in the knowledge graph. The advantage of this approach is that the database itself provides a complete graph query language and supports various graph mining algorithms. The query speed is better than that of relational databases, especially the performance of multi-hop queries.

**Therefore, our response to this comment is to add a description of the advantages of neo4j.**

**Compared with RDF storage, Neo4j offers significant advantages in terms of graph traversal performance, intuitive data modeling, real-time querying, and tool ecosystem, making it ideal for application scenarios requiring efficient graph operations and real-time responsiveness[14,15].**

**(See the 4.3 Knowledge Storage of Hypertensive Intracerebral Hemorrhage)**

Reviewer #2: This paper proposes a methodology for constructing a medical knowledge graph by integrating ontology modeling, web scraping, Unified Information Extraction (UIE), fine-tuning strategies, and graph databases. It addresses knowledge modeling, extraction, and storage techniques. The ERNIE-UIE model was fine-tuned with a small amount of annotated data, enabling the joint extraction of entities and relationships. The resulting knowledge graph comprises 8,525 entity data points and 9,522 triplet data points, with its accuracy validated using graph algorithms. The paper demonstrates that adopting a generative extraction paradigm optimizes the construction process of Chinese medical knowledge graphs, achieving commendable results in low-resource settings and offering cost-efficient solutions for knowledge graph development.

**Response:**

We feel great thanks for your professional review work on our article. As you are concerned, there are several problems that need to be addressed.According to your nice suggestions, we have made extensive corrections to our previous draft, the detailed correction are listed below.

1.The paper lacks a detailed explanation of the data sources used for web scraping and annotation. Clarifying the data selection and processing methods would enhance reproducibility and credibility.

**Response:**

Thanks for the valuable comments.The ontology construction process in this article refers to the existing OMAHA shcema in China and the Hypertension Ontology in the Open Bioinformatics Ontology (OBO) system.

**Therefore, our response to this comment was to add a description of the data source in the paper.**

**Using existing ontologies such as the Omaha Schema and the hypertension ontology within the Open Biological and Biomedical Ontology framework as a reference, an ontology model for hypertensive intracerebral hemorrhage was constructed, based on which the entity and relationship types were determined, following which the text data were labeled.**

**（See 3.1 Overall Framework)**

**The data sources used in the study were obtained via crawling the question-and-answer data from the professional medical Q&A website Dingxiang Doctor, combined with raw text data from professional literature and books sourced from China National Knowledge Infrastructure, Wanfang Medical Network, and WeChat Reading, totaling 261,494 characters.**

**（See 3.2 Data Preprocessing）**

2.The paper provides a general description of the potential applications of the knowledge graph. It would benefit from specific examples of its use in medical scenarios to better demonstrate its practical value.

**Response:**

Thanks for the valuable comments.

**Therefore, our response to this comment was to add example of using UIE technology to build knowledge graphs for public health emergency management.**

**This method holds certain potential for application in the medical field. Drawing on our previous research[18], if it is applied to knowledge extraction in the medical domain, it can provide a reliable source of medical knowledge for medical question answering and the currently popular retrieval-augmented generation research.**

**（See Discussion)**

3. The paper does not discuss potential limitations of the generative extraction paradigm, such as its performance in handling complex entity relationships or its adaptability to other domains. Adding this discussion would provide a more balanced perspective.

**Response:**

We sincerely appreciate the valuable comments.

**We have checked the literature carefully and added morereferences into the Discussion part in the revised manuscript.**

**Although the generative extraction paradigm utilized in this study demonstrated significant potential in knowledge graph construction and IE, its limitations cannot be ignored, such as its heavy reliance on the design of prompts for performance. Future research needs to address various aspects, including prompt engineering, knowledge updating, hallucination issues, complex-sentence processing, domain adaptability, multitask learning, computational resource requirements, entity disambiguation, few-shot learning, and interpretability, to further enhance the performance and practicality of the generative extraction paradigm. By integration of external knowledge sources, introduction of postprocessing steps, and designing of more sophisticated multitask learning frameworks, among other technical approaches, the generative extraction paradigm is poised to play an even greater role in the field of knowledge graphs[19,20].**

**(See Discussion)**
