# Supplementary material for: ERNIE-UIE: Advancing information extraction in Chinese medical knowledge graph
Source: PLoS One. 2025 May 29;20(5):e0325082. doi: 10.1371/journal.pone.0325082 (PMC12121792; doi:10.1371/journal.pone.0325082)
Supplement: Attachment — Submitted filename: review comment.docx [file pone.0325082.s004.docx]

The authors performed an information extraction in Chinese medical texts based on ERNIE-UIE. They expanded their work and developed an ontology model for whole-course management. The scope of work is diverse and the topic is interesting. However, the work lacks sufficient depth, with inadequate review of the most recent literature and insufficient elaboration on the innovative aspects of the research.

1. The paper suffers from inadequate literature citations, with limited investigation into extraction methodologies outside the medical domain and insufficient critical discussion of the limitations inherent in the cited medical research. There are additional works in the existing literature that should be referenced, including but not limited to:

- Ning Pang, Xiang Zhao, Weixin Zeng, Zhen Tan, Weidong Xiao. StaRS: Learning a Stable Representation Space for Continual Relation Classification. IEEE Transactions on Neural Networks and Learning Systems.

- Hei M, Zhang Z, Liu Q, et al. Enhanced Multi-Tuple Extraction for Alloys: Integrating Pointer Networks and Augmented Attention. arXiv preprint arXiv:2503.06861, 2025.

2. The paper critically lacks a clear articulation of research motivation in the Introduction section. The analysis of challenges specific to Chinese-language medical information extraction remains superficial, resulting in underdeveloped justification for the study's innovative value. Furthermore, the authors must explicitly delineate the technical distinctions between their methodology and the ERNIE-UIE framework in the Introduction; otherwise, this work risks being perceived as a straightforward implementation of ERNIE-UIE rather than a substantive methodological advancement.

3. In Section 4.1 (Model Selection), the authors merely state the use of the ERNIE-UIE model without elucidating its technical specifics. A concise overview of ERNIE-UIE's architecture and distinctive features should be provided to mitigate comprehension barriers for readers unfamiliar with this framework.
