# Supplementary material for: ERNIE-UIE: Advancing information extraction in Chinese medical knowledge graph
Source: PLoS One. 2025 May 29;20(5):e0325082. doi: 10.1371/journal.pone.0325082 (PMC12121792; doi:10.1371/journal.pone.0325082)
Supplement: Attachment — Submitted filename: Response_to_Reviewers_auresp_2.docx [file pone.0325082.s006.docx]

Response to Reviewer’s Comments

Dear Dr. Emiliano Damian Alvarez Leites and Reviewers,

We were pleased to know that our work was rated as potentially acceptable for publication in Journal, subject to adequate revision. We thank the reviewers for the time and effort that they have put into reviewing the previous version of the manuscript. Their suggestions have enabled us to improve our work. Based on the instructions provided in your letter, we uploaded the file of the revised manuscript. Accordingly, we have uploaded a copy of the original manuscript with all the changes highlighted by using the track changes mode in MS Word.

Appended to this letter is our point-by-point response to the comments raised by the reviewers. The comments are reproduced and our responses are given directly afterward in a different color.

We would like also to thank you for allowing us to resubmit a revised copy of the manuscript.

We hope that the revised manuscript is accepted for publication in the Journal of PLOS One.

Sincerely,

Jing Zheng

Journal Requirements:

1.Please review your reference list to ensure that it is complete and correct. If you have cited papers that have been retracted, please include the rationale for doing so in the manuscript text, or remove these references and replace them with relevant current references. Any changes to the reference list should be mentioned in the rebuttal letter that accompanies your revised manuscript. If you need to cite a retracted article, indicate the article’s retracted status in the References list and also include a citation and full reference for the retraction notice.

**Response:**

In accordance with the editor’s suggestions, we carefully reviewed all cited references to ensure that none of the cited works have been retracted.

2. Comments from PLOS Editorial Office: We note that one or more reviewers has recommended that you cite specific previously published works. As always, we recommend that you please review and evaluate the requested works to determine whether they are relevant and should be cited. It is not a requirement to cite these works. We appreciate your attention to this request.

**Response:**

We carefully reviewed all references suggested by the reviewers and selectively incorporated those most relevant to the content of the manuscript, in order to ensure academic rigor and the quality of citations.

Reviewer #2: The revised version has addressed my concerns.I suggest adding a discussion on the latest works in large language model-based information extraction to enhance the paper's insights. For example:

Large language models for generative information extraction: A survey

Structured information extraction from scientific text with large language models

Revisiting relation extraction in the era of large language models

IEPile: unearthing large scale schema-conditioned information extraction corpus

Autore: Document-level relation extraction with large language models

**Response:**

Thanks for your positive comments. We have extended the Introduction section to address the evolving role of large language models in information extraction, highlighting both their potential and limitations.

**With the rapid advancement of large language models (LLMs), the landscape of information extraction is undergoing a profound transformation. Unlike traditional approaches based on classification or sequence labeling, LLMs have ushered in a new paradigm known as generative information extraction, wherein extraction tasks are reformulated as text generation problems. This paradigm enables the model to produce structured outputs—such as knowledge triples—in natural language form[14].Wadhwa et al. suggest that in the era of LLMs, relation extraction is shifting from typological modeling toward open-domain, question-answering-based modeling. By leveraging natural language prompts, this approach enhances scalability and cross-domain transferability[15].Nevertheless, generative extraction approaches continue to grapple with challenges such as inconsistent outputs and the omission of multi-entity or complex relational structures. To address these limitations, recent studies have advocated for the use of predefined schemas or domain ontologies to guide model outputs, thereby enforcing consistency and completeness. For instance, schema-conditioned extraction frameworks utilize structured templates to constrain and inform generation, offering a promising pathway toward building high-quality, domain-specific knowledge graphs[16].**

**（see Introduction line 96-110 )**

Reviewer #3: This paper presents ERNIE-UIE, a method for extracting information from Chinese medical texts to build knowledge graphs with minimal annotated data. The research team fine-tuned the ERNIE-UIE model using just 200 annotated entries to extract entities and relationships from medical texts about hypertensive intracerebral hemorrhage.

1.The paper would benefit from a more detailed technical description of the prompt construction process for ERNIE-UIE. While the authors mention that prompts follow the format of "head entity's relationship type" and briefly explain the Structural Schema Instructors (SSI) concept, it may be helpful to provide sufficient details about prompt optimization for the medical domain.

**Response:**

Thanks for the valuable comments.

The corresponding details are presented in both **3.2** **data preprocessing section (line 222-236)and Section 4.2 Joint Entity-Relationship Extraction of the manuscript(358-389).**

2.It would be useful to state what specific procedures were used to handle medical entity disambiguation during graph construction, and how the authors addressed connectivity issues in the knowledge graph.

**Response:**

Thanks for the valuable comments.We have acknowledged in the **Discussion section** that a limitation of this study is the lack of entity disambiguation, which remains an important issue to be addressed in future work. We sincerely appreciate the reviewer’s insightful comments regarding this point.

**However, the model poorly performed for certain user-defined entity types and relationship types (see Tables 3 and 4). This may be because it is a pretrained language model, because of which although it retains the capability to extract general information from large-scale data during pretraining, it is not ideal for extraction of long phrases or sentences that lack typical sentence patterns and grammatical features. After fine-tuning, some metrics continued to exhibit poor performance and there was no entity disambiguation for the knowledge extracted, resulting in some semantically similar entities within the final knowledge set.**

**（see Discussion line 542-549)**

**In response, additional content has been incorporated to clarify how connectivity challenges within the knowledge graph were resolved.**

**In this study, the ERNIE-UIE model was employed for knowledge extraction, and several key characteristics of the model are noteworthy. First, the model adopts a prompt-based strategy for triple extraction, in which the prompt serves as the input and is constructed using the head entity and the relation in a natural language-compliant format, such as "A's B" or the genitive form "B of A"—for instance, "intervention for disease." This design enables the generated prompt to align closely with linguistic intuitiveness and semantic clarity.Second, the output of the model—formatted as a JSON file—contains detailed information about the head entity (including its content and entity type), the relation type, and the tail entity content. However, it lacks information regarding the entity type of the tail entity.To address these issues, the semantic compatibility between the head entity and the relation was carefully considered during prompt construction, particularly in terms of the noun–verb collocational semantics, to ensure that the generated prompts retain meaningful interpretation and thereby enhance extraction performance. In addition, to compensate for the absence of tail entity type annotations, a strategy based on unique relation types was implemented. This approach allows for the post hoc inference of the tail entity type by mapping the extracted results according to predefined head–relation–tail structure patterns, wherein each unique relation reliably corresponds to a specific tail entity type.**

**(see 4.2 Joint Entity-Relationship Extraction line 329-344)**

3.The authors should expand their literature review to include cutting-edge techniques like GraphRAG and LightRAG, which represent significant advancements in combining retrieval-augmented generation with graph-based knowledge structures.

**Response:**

We appreciate your valuable suggestion. **In response, we have incorporated additional discussion on research related to retrieval-augmented generation (RAG).**

**Notably, in recent natural language processing (NLP) research, retrieval-augmented generation (RAG) has emerged as a widely adopted framework that integrates generative language models with external knowledge retrieval. This approach has demonstrated considerable utility across a range of complex tasks, including multi-hop question answering, information extraction, and knowledge graph construction. As the complexity of application scenarios continues to grow, research within the RAG paradigm has begun to shift from unstructured document retrieval toward more structured, controllable, and interpretable forms of knowledge integration. Of particular interest is the incorporation of graph-structured knowledge, as exemplified by recent frameworks such as GraphRAG and LightRAG.GraphRAG represents a notable advancement in this area by combining retrieval-augmented generation with knowledge graph reasoning. It transforms retrieved documents into entity-relation graphs and applies graph neural networks (GNNs) to enhance the model’s capacity for structural semantic understanding and logical inference during text generation. Empirical evidence has shown that this method significantly improves answer consistency and factual accuracy on challenging multi-hop tasks such as HotpotQA [27].**

**However, GraphRAG faces practical challenges, including high graph construction overhead and deployment complexity. In response, lightweight alternatives such as LightRAG have been introduced. LightRAG simplifies the graph structure into shallow entity connectivity graphs and incorporates efficient retrieval indexing techniques, thereby maintaining comparable performance while substantially reducing inference latency and resource consumption [28].To further advance the integration of generative models with structured information, recent studies have developed resources such as IEPile—a schema-conditioned information extraction corpus designed to support transfer learning and capability evaluation of large language models in structured output tasks across multiple domains[16].**

**In addition, recent studies have explored the incorporation of graph-structured constraints directly into the decoding process of retrieval-augmented generation (RAG) frameworks. Recent studies have explored the direct incorporation of structural graph constraints into the decoding process of retrieval-augmented generation (RAG) frameworks. For instance, GraphRAFT—a retrieval and reasoning framework—fine-tunes large language models (LLMs) to generate verifiably correct Cypher queries, thereby retrieving high-quality subgraph contexts and enabling the production of accurate, evidence-based answers. This approach leverages the synergy between structured knowledge retrieval and generative reasoning, addressing both the reliability and precision of knowledge-intensive outputs[29].In medical settings, certain approaches have sought to integrate specialized knowledge graphs to augment the performance of large language models (LLMs). For example, MedGraphRAG leverages a medical knowledge graph—customized specifically for processing private healthcare data—to enhance the capabilities of LLMs[30].Collectively, the integration of graph-structured knowledge into RAG architectures has significantly enhanced the model’s capabilities in structural recognition, factual reasoning, and semantic consistency. Future research may further investigate adaptive knowledge graph construction, cross-task representation sharing of graph structures, and interpretable graph-based inference. These directions hold substantial promise for expanding the utility of graph-enhanced RAG models in large-scale question answering and knowledge extraction tasks.**

**(see Discussion line 560-601)**

Reviewer #4:The authors performed an information extraction in Chinese medical texts based on ERNIE-UIE. They expanded their work and developed an ontology model for whole-course management. The scope of work is diverse and the topic is interesting. However, the work lacks sufficient depth, with inadequate review of the most recent literature and insufficient elaboration on the innovative aspects of the research.

1. The paper suffers from inadequate literature citations, with limited investigation into extraction methodologies outside the medical domain and insufficient critical discussion of the limitations inherent in the cited medical research. There are additional works in the existing literature that should be referenced, including but not limited to:

- Ning Pang, Xiang Zhao, Weixin Zeng, Zhen Tan, Weidong Xiao. StaRS: Learning a Stable Representation Space for Continual Relation Classification. IEEE Transactions on Neural Networks and Learning Systems.

**Response:**

Thanks for the valuable comments.**We acknowledge the shortcomings in our literature review and have accordingly supplemented this section to address the gaps.**

**In the field of materials science, Hei et al. proposed a novel framework integrating pointer networks and augmented attention mechanisms to extract complex multi-tuple relations from scientific literature, particularly those pertaining to alloy mechanical properties[11]. Their approach was designed to overcome the limitations of traditional methods in handling nested entities, contextual ambiguity, and one-to-many relational mappings. However, unlike materials science, medical informatics presents additional challenges, including higher annotation costs and a more dynamic data landscape—particularly in response to emerging disease variants. These characteristics necessitate the development of models with enhanced transfer learning capabilities.To address continual relation classification, Pang et al.introduced a strategy that combines knowledge distillation with contrastive learning to mitigate the problem of catastrophic forgetting. While effective in general domains, their method did not account for the unique temporal dynamics of medical data, such as the frequent updates to clinical guidelines and evolving diagnostic criteria [12].**

**（see introduction line 66-78)**

2. The paper critically lacks a clear articulation of research motivation in the Introduction section. The analysis of challenges specific to Chinese-language medical information extraction remains superficial, resulting in underdeveloped justification for the study's innovative value. Furthermore, the authors must explicitly delineate the technical distinctions between their methodology and the ERNIE-UIE framework in the Introduction; otherwise, this work risks being perceived as a straightforward implementation of ERNIE-UIE rather than a substantive methodological advancement.

**Response:**

Thanks for the valuable comments.**To address this shortcoming, we have incorporated additional literature in the Introduction.**

**In the development of Chinese medical information extraction, scholars have made continuous progress while simultaneously uncovering a range of challenges unique to this domain. One major linguistic obstacle arises from the absence of natural word boundaries in the Chinese language, which frequently leads to incorrect segmentation of clinical terms during the tokenization stage. Such segmentation errors can propagate through downstream tasks—most notably named entity recognition—resulting in cascading inaccuracies. For example, the phrase “高血压病史三年” (a three-year history of hypertension) should be recognized as a single unit representing a medical condition. However, if it is incorrectly segmented into “高/血压/病史” (high / blood pressure / history), the semantic integrity of the expression is easily compromised.Moreover, in contrast to English-language biomedical text mining—which benefits from well-established ontologies such as UMLS and SNOMED CT—the Chinese context still largely relies on manually curated lexicons or domain-specific knowledge graphs, such as CMKG [13]. These resources often exhibit limitations in terms of coverage, connectivity, and the degree of structural formalization.In addition, the sensitive and regulated nature of medical data presents a further obstacle, significantly restricting access to high-quality, publicly available corpora and annotated samples. Consequently, Chinese medical information extraction faces multifaceted challenges spanning linguistic representation, knowledge modeling, and task-level robustness.**

**(see Introduction line 79-95)**

**Building upon the ERNIE-UIE framework, this study incorporates a schema grounded in established medical ontologies to address domain-specific challenges inherent in medical information extraction. To enhance the coverage and diversity of input data, medical texts were expanded through automated web-crawling techniques. Furthermore, a unique relation-mapping strategy was introduced to resolve instances where ERNIE-UIE fails to generate complete relational tuples, particularly with missing tail entities. Collectively, this work establishes a transferable and systematic methodology that extends the utility of ERNIE-UIE-based models for medical knowledge extraction, knowledge graph construction, and downstream clinical applications.**

**(see Introduction line 130-138)**

3. In Section 4.1 (Model Selection), the authors merely state the use of the ERNIE-UIE model without elucidating its technical specifics. A concise overview of ERNIE-UIE's architecture and distinctive features should be provided to mitigate comprehension barriers for readers unfamiliar with this framework.

**Response:**

Thank you for the insightful comment. **Accordingly, we have supplemented the model selection section with a brief overview of the model.**

**The model employed in this study is ERNIE-UIE, a unified information extraction framework developed based on UIE by PaddleNLP. It utilizes ERNIE 3.0—a knowledge-enhanced large-scale language model within the Wenxin (文心) foundation model family—as its encoder, and adopts a dual-pointer decoding architecture. ERNIE-UIE is a general-purpose Chinese information extraction model trained through large-scale multi-task learning.By leveraging universal extraction capabilities acquired via extensive multitask pretraining, alongside a prompt-based unified modeling approach for information extraction tasks, ERNIE-UIE demonstrates robust adaptability across a wide array of domains and extraction objectives. Notably, the model supports zero-shot initialization, enabling rapid deployment without prior task-specific annotation. Furthermore, it exhibits strong few-shot fine-tuning performance, achieving substantial improvements in domain-specific information extraction even with limited annotated data.**

**(see 4.1 Model Selection line 268-279)**
